# Supplementary material for: Lateral melt variations induce shift in Io’s peak tidal heating
Source: Nat Commun. 2025 Jul 23;16:6798. doi: 10.1038/s41467-025-62059-4 (PMC12287347; doi:10.1038/s41467-025-62059-4)
Supplement: Supplementary file 1 — Supplementary Information [file 41467_2025_62059_MOESM1_ESM.pdf]

Lateral Melt Variations induce Shift in Io's Peak Tidal Heating

Allard Veenstra[1*], Marc Rovira-Navarro[1], Teresa Steinke, Ashley Gerard Davies[2], Wouter van der Wal[1]

[1*]Faculty of Aerospace Engineering, TU Delft, Building 62 Kluyverweg 1, Delft, 2629 HS, The Netherlands.

[2]Jet Propulsion Laboratory, California Institute of Technology, Pasadena, CA, USA.

*Corresponding author(s). E-mail(s): a.k.veenstra@tudelft.nl;
Contributing authors: m.roviranavarro@tudelft.nl; ;
Ashley.Davies@jpl.nasa.gov; w.vanderwal@tudelft.nl;

Supplementary Figures

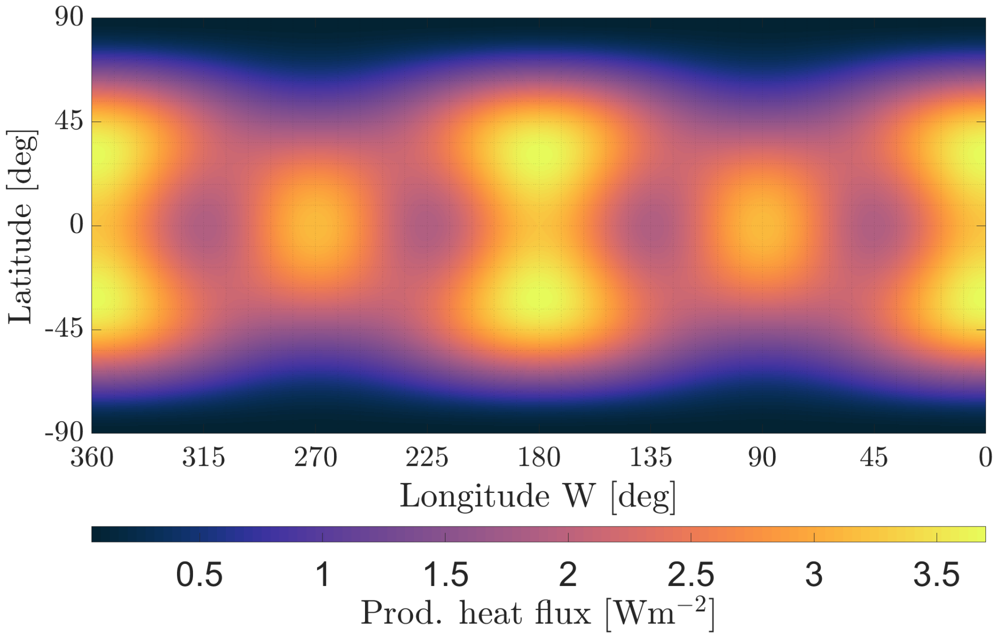

Supplementary Fig. 1: Surface heat flux for a spherically symmetric Io with solid-body dissipation in the asthenosphere. The map is centered on the anti-subjovian point.

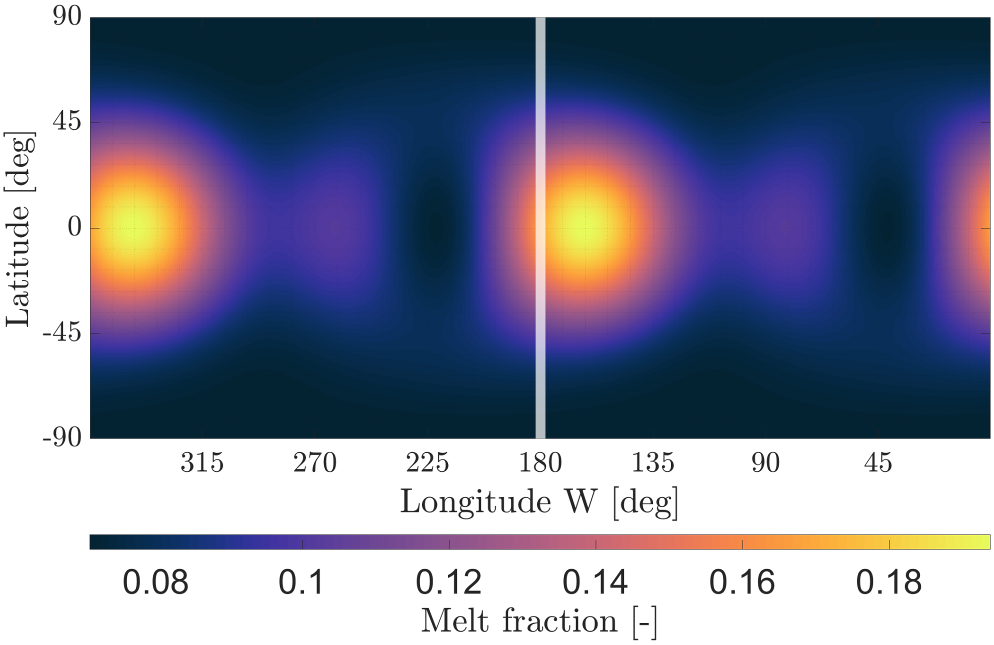

Supplementary Fig. 2: Melt fraction resulting from, and causing, the tidal dissipation pattern presented in Fig. 2. The vertical grey line indicates 180° W.

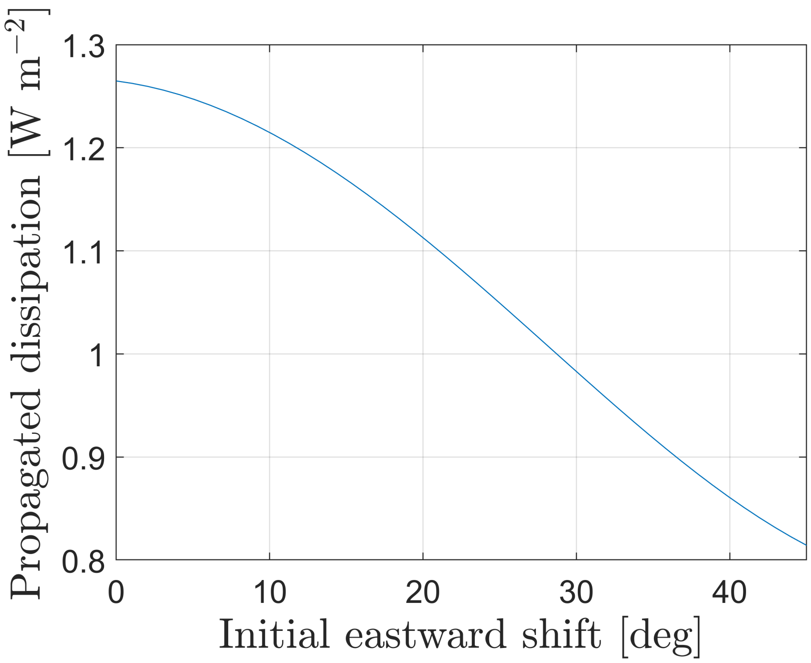

Supplementary Fig. 3: The propagated (degree and order 2) mode of the tidal dissipation spectrum used in the toy model. Plotted as a function of the eastward shift of the input melt fraction pattern. The input melt fraction pattern that was used to create this plot has peak-to-peak variations of 86%.

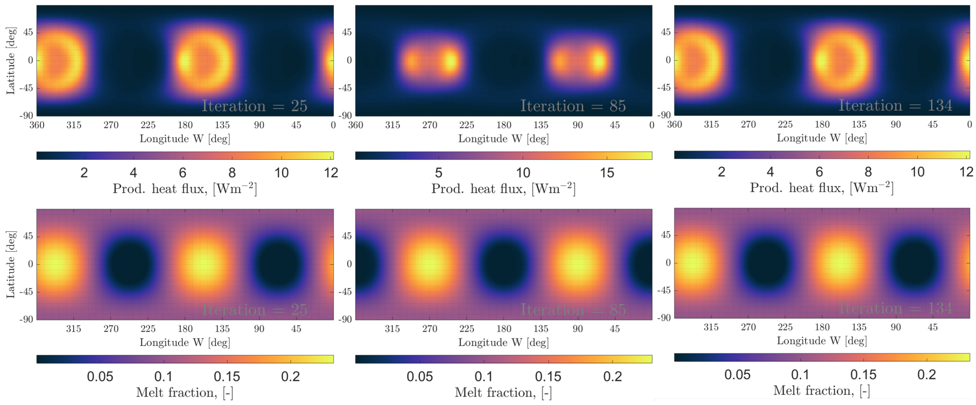

Supplementary Fig. 4: Alternating tidal and heat flux patterns that arise for a very strong coupling in the toy model. The top plots show the surface heat flux, and the bottom plots show the melt fraction patterns at different iterations. The plots demonstrate that when c becomes too large, there is no longer a stable pattern, but that the system repeats between different patterns while the peak moves continuously eastwards.

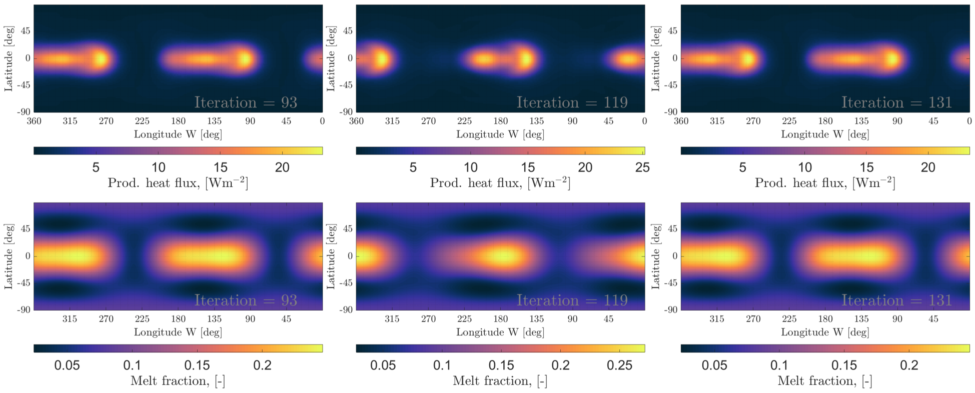

Supplementary Fig. 5: Similar plot to Supplementary Fig. 4 but now for the full model.

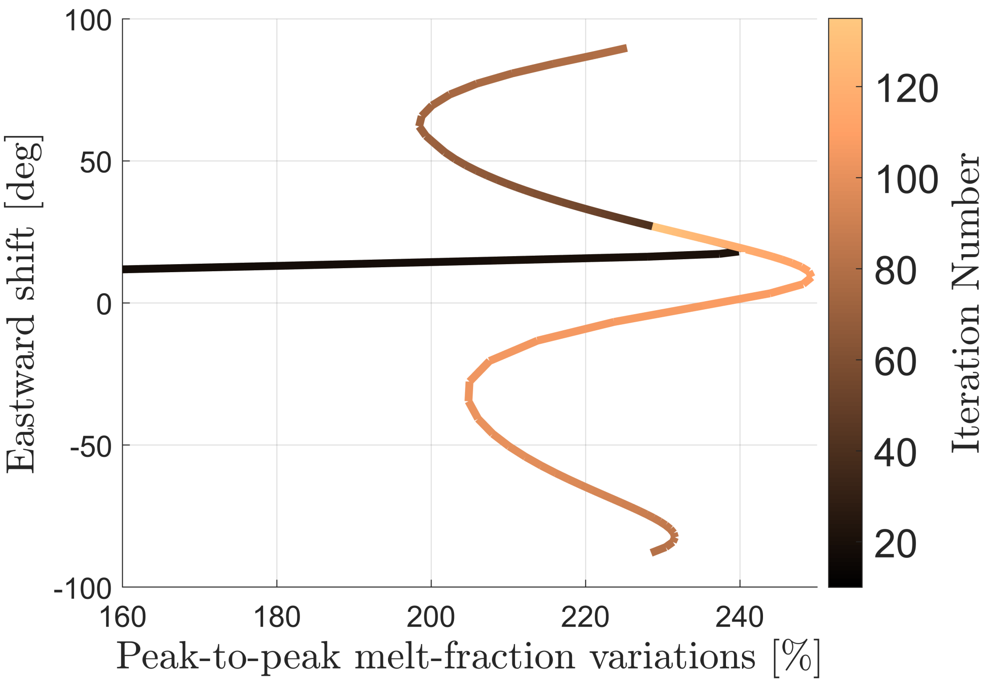

Supplementary Fig. 6: The trajectory of the melt fraction pattern, plotted in the same parameter space as Fig. 3, for a very strong coupling. The eastward shift with respect to the prime meridian of the degree and order 2 melt fraction mode (same as the initial eastward shift in Fig. 3) is plotted against the peak-to-peak melt fraction variations. The color of the line represents the number of iterations.

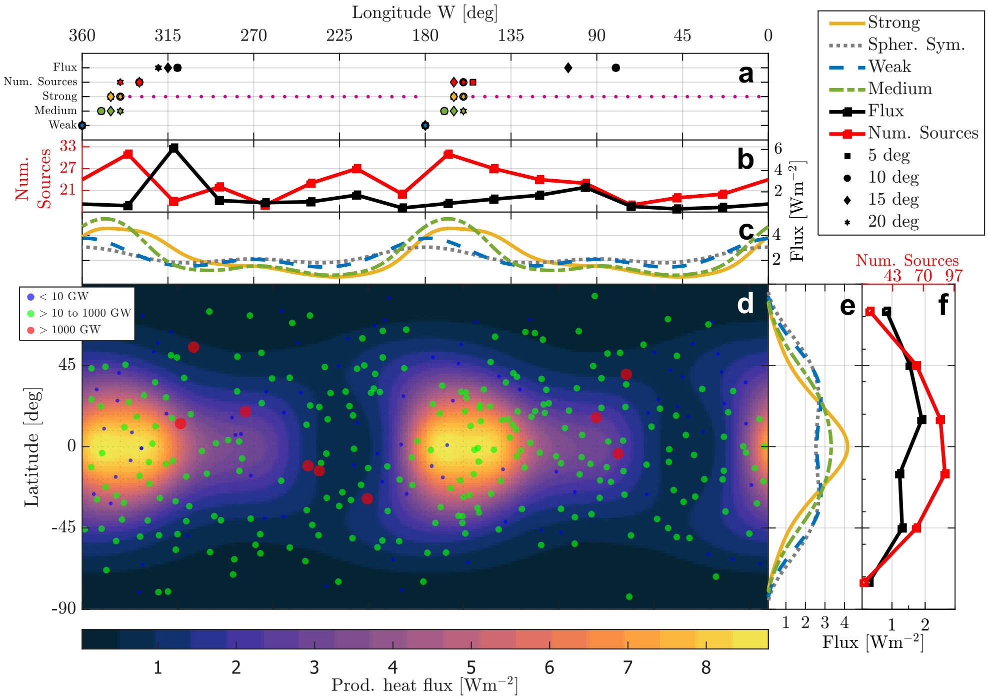

Supplementary Fig. 7: A plot similar to Fig. 2 but created by normalizing the tidal dissipation. The weak, medium, and strong lines correspond to $c = 0.0075$, $c = 0.0125$, and $c = 0.015$ respectively. The values for c are chosen such that the plot looks very similar to Fig. 2.

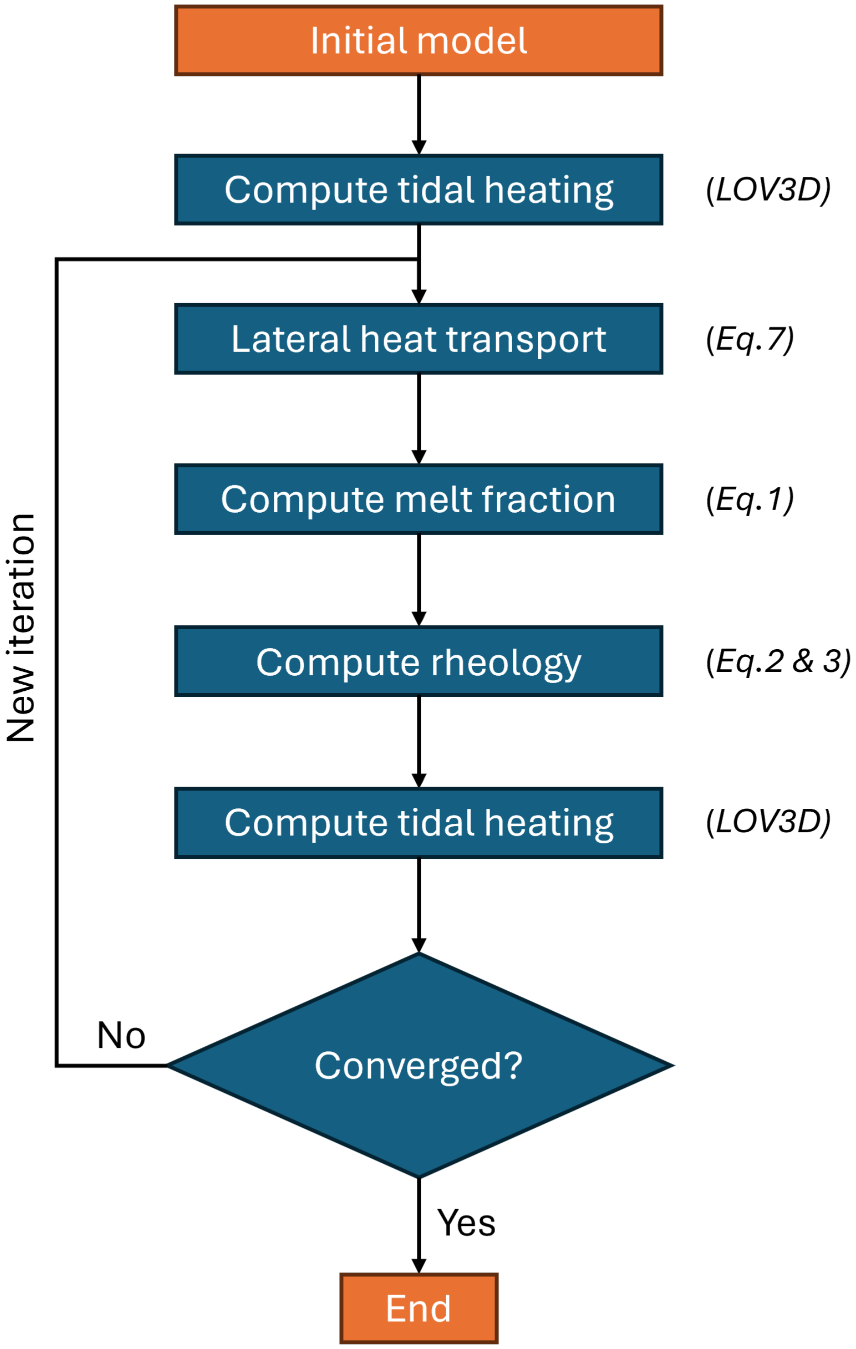

Supplementary Fig. 8: Schematic of the different steps in the iteration of the feedback mechanism. The equation numbers correspond to the relevant equations in the Method.

Supplementary Tables

Supplementary Table 1: Spearman rank correlations between different aspects of the observations and the spherically symmetric model, a model with a strong coupling strength ($c = 0.0105$), and a model with a medium coupling strength ($c = 0.00975$). The bins are located such that the centre of the last bin is at 360°W, changing the bin spacing will change the absolute correlations but not the relations between models. Compared to Fig. 2, we have removed the bin sizes smaller than 15 due to a mismatch in the spatial frequency content between the data and the model. The observations are a collection of point sources that have a high variability on small scales. The model data, on the other hand, only simulates long-wavelength features. For the strong coupling case, there is only significant dissipation up to spherical harmonic degree 12 (equivalent to a bin size of 15 degrees), but most of the energy is found at degrees 2, 4, and 6. Comparing the model to the observations at these small bin sizes is therefore not very representative of any actual correlation.

Bin sizes [deg]:		15	20	36
2D Heat flux distribution	Spherically symmetric	0.389	0.313	0.250
	Medium coupling	0.406	0.327	0.227
	Strong coupling	0.428	0.351	0.248
2D Distribution of features	Spherically symmetric	0.506	0.592	0.744
	Medium coupling	0.542	0.606	0.771
	Strong coupling	0.564	0.630	0.791
Longitudinal distribution of features	Spherically symmetric	-0.167	-0.148	0.039
	Medium coupling	0.049	0.235	0.469
	Strong coupling	0.281	0.343	0.469

Supplementary Discussion

Comparison with a FEM model

Here, we compare the output of LOV3D with a FEM model [1]. The FEM model is built in ABAQUS using the method described in Wu [2] to account for self-gravitation. The model was initially developed by Hu et al. [3] and later modified by Steinke et al. [1] to apply it to Io. The runs shown here use a grid size of 1° and are split into 19 radial layers: 6 comprising the mantle, 10 for the asthenosphere, and 3 for the lithosphere [4]. The total run time was 4 orbits with 15 timesteps per orbit, and only the last orbit was used to compute the orbital-average tidal heating.

In the comparison, we introduce lateral variations of tidal heating of spherical harmonics degree 2 expected from asthenospheric heating (pattern B in Beuthe [5]) and map them to melt fraction variations as described in the Method and Steinke [4] using

$$\delta\Phi(\theta,\phi) = c\, Q_{\mathrm{ref,obs}} \left(\frac{\Psi_2(\theta,\phi)}{\Psi_0}\right) \ ,$$

with $c = 0.02$, $Q_{\mathrm{ref,obs}} = 2.3$ Wm^{-2}, $\Psi_0 = 21/5$, and

$$\Psi_2(\theta,\phi) = 0.5\left(-\frac{33}{7}P_{20}(\cos\theta) + \frac{9}{14}P_{22}(\cos\theta)\cos(2\phi)\right),$$

with $P_{20}(\cos\theta)$ and $P_{22}(\cos\theta)$ the degree 2 associated Legendre polynomial of order 0 and 2 respectively. The average melt fraction is 10%. We compute the tidal dissipation with this pattern as input, shown in a) of Supplementary Fig. 9, or the same pattern but shifted eastward by 30 degrees, shown in b) of Supplementary Fig. 9. Both codes used the same interior properties (given in Table **??**) and used $B_\eta = 20$ instead of the $B_\eta = 26$ that was used to create the main results of the article.

We found that the mean heat flux predicted by the FEM model is consistently lower than with *LOV3D*. This is also the case for the spherically-symmetric case, for which

the spectral model provides the expected average dissipation from semi-analytical models and the FEM underestimates it by roughly 20%. The underestimation of tidal heating in the FEM models is the result of the coarse model resolution, which cannot capture strong radial gradients in tidal heating in the thin asthenosphere, and the relatively short spin-up phase of the FEM, both required to keep the numerical cost at bay.

As these numerical problems are expected to affect the outcome of both the spherically symmetric model and the one with lateral variations in a similar way, the effect of lateral variations can be better compared using the heat flux normalized with respect to the mean. Doing so, we find excellent agreement between the predictions of the FEM model and *LOV3D*. The output of *LOV3D* is plotted in the top row and since the results are very similar, we do not plot the output of the FEM model but rather the difference between the output of the two codes in the bottom row. What is left is clearly a result of the discretization in the FEM model. The pattern and relative magnitude of tidal heating anomalies caused by lateral variations agree, showing discrepancies of around 5%.

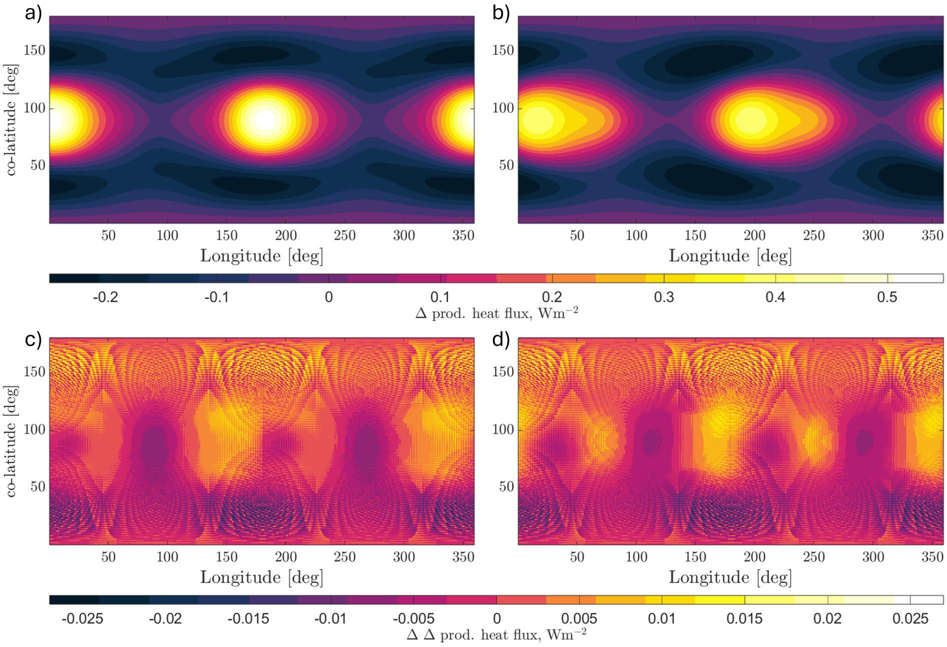

Supplementary Fig. 9: Normalized difference in tidal dissipation of an Io with lateral variations compared to that of a uniform Io with LOV3D and a spectral code. The plots in the top row (a,b) were generated using *LOV3D* and the ones in the bottom row (c,d) are the difference of the top row with respect to the same results generated using a FEM code [4]. The lateral variations used in the plots of the left column (a,c) are centered at the prime meridian while those in the right column (b,d) have their initial melt-fraction pattern shifted eastwards by 30 degrees.

References

[1] Steinke, T., Hu, H., Höning, D., van der Wal, W., Vermeersen, B.: Tidally induced lateral variations of Io's interior. Icarus **335**, 113299 (2020) https://doi.org/10.1016/j.icarus.2019.05.001

[2] Wu, P.: Using commercial finite element packages for the study of earth deformations, sea levels and the state of stress. Geophysical Journal International **158**(2), 401–408 (2004) https://doi.org/10.1111/j.1365-246X.2004.02338.x

[3] Hu, H., Wal, W., Vermeersen, L.L.A.: A numerical method for reorientation of rotating tidally deformed viscoelastic bodies. Journal of Geophysical Research: Planets **122**(1), 228–248 (2017) https://doi.org/10.1002/2016JE005114

[4] Steinke, T.: The Curious Case of Io - Connections Between Interior Structure, Tidal Heating and Volcanism. PhD thesis, Delft University of Technology (2021). Chap. 3. https://doi.org/10.4233/uuid:9e875752-05bc-4dd8-9bdd-77e18cf3c43f

[5] Beuthe, M.: Spatial patterns of tidal heating. Icarus **223**(1), 308–329 (2013) https://doi.org/10.1016/j.icarus.2012.11.020
